# 6 weeks of fartlek-style endurance training does not alter physiological responses to mild cold exposure but attenuates the cold-induced plasma noradrenaline response: A randomized, matched control trial

**DOI:** 10.1016/j.jcte.2026.100455

**Published:** 2026-08-25

**Authors:** Alexander Braunsperger, Lara Becker, David P. Camargo, Sidney Radsak, Magdalena Paintner, Dimitrios Kossenakis, Xianyu Song, Anna Kraus, Alina Birnkammer, Sebastian Mesmer, Joshua Stengel, Lukas Frank, Svenja Kuger, Tobias Mather, Enni Peltola, Marcel Könen, Kirsten Liebetanz, Jule Zorn, Meri De Angelis, Martin Hrabe de Angelis, Dominik Tischer, Ana Soriano-Arroquia, Anastasia Georgiadi, Martin Schönfelder, Henning Wackerhage

**Affiliations:** a Professorship of Exercise Biology, Department Health and Sport Sciences, TUM School of Medicine and Health, Technical University of Munich, Munich, Germany; b Institute for Diabetes and Adipositas, Helmholtz Munich, German Center for Diabetes Research (DZD), Neuherberg, Germany; c Institute of Experimental Genetics and German Mouse Clinic, Helmholtz Center Munich, German Research Center for Environmental Health, Neuherberg, Germany; d Institute for Pharmacology and Toxicology, Biomedical Center, University of Bonn, Bonn, Germany; e Institute for Diabetes and Cancer, Helmholtz Munich, German Center for Diabetes Research (DZD), Neuherberg, Germany; f Chair of Experimental Genetics, School of Life Science Weihenstephan, Technische Universität München, Freising, Germany; g German Center for Diabetes Research (DZD), Neuherberg, Germany

**Keywords:** Exercise training, Cold exposure, Energy metabolism, Brown adipose tissue

## Abstract

**Background and aims:**

Rodent studies suggest that endurance training may promote the browning of white adipose tissue and enhances adipose tissue thermogenesis during cold exposure. However, it remains unclear whether endurance training leads to similar adaptations in humans. The aim of this study was therefore to find out whether six weeks of intensive, fartlek-style cycle endurance training alters responses to cold exposure, specifically cold-induced changes of oxygen uptake, plasma noradrenaline concentrations and the effect of conditioned serum on markers of adipocyte browning in culture.

**Methods and results:**

Twenty-four healthy, untrained participants completed a six-week fartlek-style cycling intervention (3 sessions/week) or control period. We estimated cold-induced adaptive thermogenesis before and after the intervention by indirect calorimetry during 60 min of mild cold exposure at 18 °C. Endurance training increased on average the V˙O₂max by 5 ml/kg/min (95% CI 1 to 8; *p* = 0.010), maximal power output by 19 W (95% CI 5 to 33; *p* = 0.011), and power at the 4 mmol/l lactate threshold by 30 W (95% CI 18 to 43; *p* < 0.001). However, training did not alter cold-induced oxygen uptake, carbon dioxide production, energy expenditure, supraclavicular skin temperature, or mean skin temperature compared with controls. In an exploratory analysis, the cold-induced increase in plasma noradrenaline was attenuated after endurance training (−176 pg/ml, 95% CI -337 to −14; *p* = 0.035), whereas the exercise-induced noradrenaline response not altered significantly. Cold- or exercise-conditioned serum did not alter *UCP1* mRNA expression in human beige adipocytes.

**Conclusion:**

Six weeks of fartlek-style endurance training increased cardiorespiratory and metabolic fitness but had little effect on the response to cold exposure with no evidence for adipose tissue browning based on the conducted serum incubation experiment.

## Highlights

• 6 weeks of thrice weekly fartlek-style cycling endurance training induces strong cardiorespiratory and metabolic adaptations.
• Endurance training does not increase the oxygen uptake response to mild cold exposure nor the ability of serum to stimulate *UCP1* expression in adipocytes.
• Endurance training attenuated the cold-induced plasma noradrenaline response.

## Introduction

Humans have white adipose tissue (WAT), heat-generating brown adipose tissue (BAT) and beige adipose tissue which develops especially in response to cold exposure. Researchers became interested in BAT and beige adipose tissue because activated BAT in humans increases energy expenditure [1], [2], [3], [4] and its presence is associated with better metabolic health such as a lower prevalence of diabetes and obesity [5], [6], [7]. Moreover, white adipose tissue can “brown” to develop into beige adipose tissue, marked by expression of the thermogenic gene *UCP1*.

Over the past decade many researchers have investigated whether exercise training activates brown BAT thermogenesis and whether it stimulates WAT browning [8], [9], [10], [11]. Interest in this goes back to 2012 when irisin was discovered as an exercise- and PGC-1α-induced myokine that promoted *Ucp1* expression and WAT browning in mice [12]. Several subsequent studies mainly in mice identified other exercise or cold exposure-induced regulators of adipose tissue thermogenesis such as irisin, its precursor Fndc5, fibroblast growth factor FGF21, meteorin-like hormone (metrnl), prosaposin, β-aminoisobutyric acid (BAIBA), 12-lipoxygenase (12-LOX) and 12, 13 dihydroxy-9Z-octadecenoic acid (12,13-diHOME) [12], [13], [14], [15], [16], [17], [18], [19].

However, it is challenging to investigate the effect of exercise on BAT activity and white adipose tissue browning in humans. This is because ^18^fluordeoxyglucose positron emission tomography (^18^F-FDG PET) requires radioactive tracers, because there are many adipose tissue depots in humans, and because adipose tissue biopsies are invasive. The most advanced randomized control trial addressing the question of whether endurance training leads to BAT activation is the so called ACTIBATE study. In this study the investigators performed ^18^F-FDG PET scans in 35 controls, 31 participants who engaged in moderate endurance training and 31 participants who engaged in vigorous endurance training over 24 weeks. The researchers found no difference in BAT volume or ^18^F-fluorodeoxyglucose uptake and concluded that there was no evidence of an exercise-induced change on BAT volume or activity in young sedentary adults [20]. This seems surprising because BAT thermogenesis and WAT browning regulators such as noradrenaline [21], [22] or inosine [23], [24] increase during acute endurance exercise. Whilst this has demonstrated that human BAT volume or BAT glucose uptake does not respond to combined resitance and endurance training, it remains unclear whether WAT browning and energy expenditure which is influenced by BAT and beige adipose tissue thermogenesis as well as shivering adapt to endurance exercise in humans.

Thus, we designed a randomized control trial to find out whether endurance training leads to adaptations that affect the oxygen uptake and energy expenditure response to cold exposure as well as induced regulators (e.g. noradrenaline) of white adipose tissue browning in serum. Our primary aim was to investigate the effect of six weeks of fartlek-style cycle endurance training on the in vivo response to mild cold exposure (i.e., 60 min at 18 °C), which is known to activate BAT thermogenesis [3], [25], [26], [27], but also increases skeletal muscle shivering [28], [29], [30]. The second aim was to compare plasma levels of noradrenaline, the classical activator of thermogenesis, after both exercise and cold exposure before and after 6-weeks of thrice weekly endurance exercise. The third aim was to investigate the effect of conditioned serum on cultured, differentiated human beige adipocytes by assessing *UCP1* gene expression as a marker of browning. To further investigate the effects of exercise-conditioned serum on beige adipocytes, we assessed catecholamine-stimulated intracellular cAMP levels and lipolysis as markers of β-adrenergic signaling and adipocyte lipolytic responsiveness.

## Methods

### Ethical approval and study registration

All experimental procedures of this study were approved by the ethical committee of the Technical University of Munich. The registration number of the study is “2021-462_4-S-NP”. This study was retrospectively registered at ClinicalTrials.gov (NCT07299604) since participant recruitment and data collection were completed before authors decided on the journal submission. The target journal set requirements regarding trial registration and therefore this study was retrospectively registered. The primary outcomes and analysis plan were defined before the start of the intervention study and subsequent data analysis.

### Participants

A total of 24 untrained and healthy participants were recruited and screened during online interviews during the months between December 2025 and February 2026 for eligibility to be involved in a six-week, fartlek-style endurance training intervention. The pre-tests were conducted from February to April and the post-tests from April to June. As a result, matched participant pairs began the six-week intervention on a rolling basis throughout this period. Inclusion criteria were a BMI between 18 and 25 and less than 3 h per week of regular exercise training. Exclusion criteria included cardiovascular, pneumological, neoplastic, orthopedic, metabolic, chronic diseases or problems that preclude endurance performance testing. Signed informed consent for both the study participation as well as the publication of all the collected information in an open access publication was obtained. Participants were randomly allocated into training (*n* = 12) or control group (*n* = 12) in a sex-matched control design. L.B. used a central computer to pair-match participants by sex and randomly allocated matched participants to the control or training group. Each group consisted of 6 women and 6 men, respectively. Table 1 shows the participant's characteristics before and after the intervention. Matched controls performed pre- and post-tests during the same calendar week to account for confounders such as e.g., study participation bias and increasing outside temperatures. All tests were performed between 7 and 12 AM on each of the test days, in the same rooms under similar conditions (room temperature: 20–22 °C; room humidity: 20–40%).

**Table 1:** Participant characteristics (*n* = 24).

	Training group (*n* = 12)		Control group (*n* = 12)	
Characteristic	Pre-test	Post-test	Pre-test	Post-test
Age (years)	27 ± 4	27 ± 4	23 ± 3	23 ± 3
Body weight (kg)	65 ± 7	65 ± 7	67 ± 8	66 ± 8
Height (cm)	170 ± 7	170 ± 7	172 ± 11	172 ± 11
Body fat (%)	21.0 ± 3.8	20.7 ± 3.7	24 ± 8.5	22.4 ± 9.0
BMI (kg/m^2^)	22.3 ± 1.5	22.4 ± 1.4	22.5 ± 1.7	22.3 ± 1.5
Self-reported PA per week (h)	1.6 ± 1.0	nA	1.9 ± 1.2	2.5 ± 1.8
	**Men (*n* = 6)**		**Men (*n* = 6)**	
**Characteristic**	**Pre-test**	**Post-test**	**Pre-test**	**Post-test**
Age (years)	28 ± 4	28 ± 4	22 ± 2	22 ± 2
Body weight (kg)	71 ± 5	71 ± 5	72 ± 3	71 ± 5
Height (cm)	174 ± 6	174 ± 6	179 ± 7	179 ± 7
Body fat (%)	19.0 ± 2.8	18.5 ± 3.3	18.6 ± 6.8	16.2 ± 6.5
BMI (kg/m^2^)	23.3 ± 1.3	23.3 ± 1.2	22.4 ± 1.6	22.1 ± 1.7
Self-reported PA per week (h)	1.4 ± 1.1	nA	1.3 ± 1.2	3.4 ± 2.3
	**Women (*n* = 6)**		**Women (*n* = 6)**	
**Characteristic**	**Pre-test**	**Post-test**	**Pre-test**	**Post-test**
Age (years)	26 ± 5	26 ± 5	24 ± 3	24 ± 3
Body weight (kg)	59 ± 3	60 ± 3	61 ± 8	61 ± 8
Height (cm)	167 ± 7	167 ± 7	164 ± 9	164 ± 9
Body fat (%)	23.0 ± 3.7	22.9 ± 2.8	29.4 ± 6.6	28.6 ± 6.6
BMI (kg/m^2^)	21.3 ± 0.7	21.5 ± 1.0	22.6 ± 1.9	22.5 ± 1.7
Self-reported PA per week (h)	1.7 ± 1.0	nA	2.4 ± 1.0	1.7 ± 0.6
Values expressed as mean ± SD.				

Abbreviations: BMI, body mass index; PA, physical activity; nA, non-applicable.

### Experimental design

**Fig. 1:**
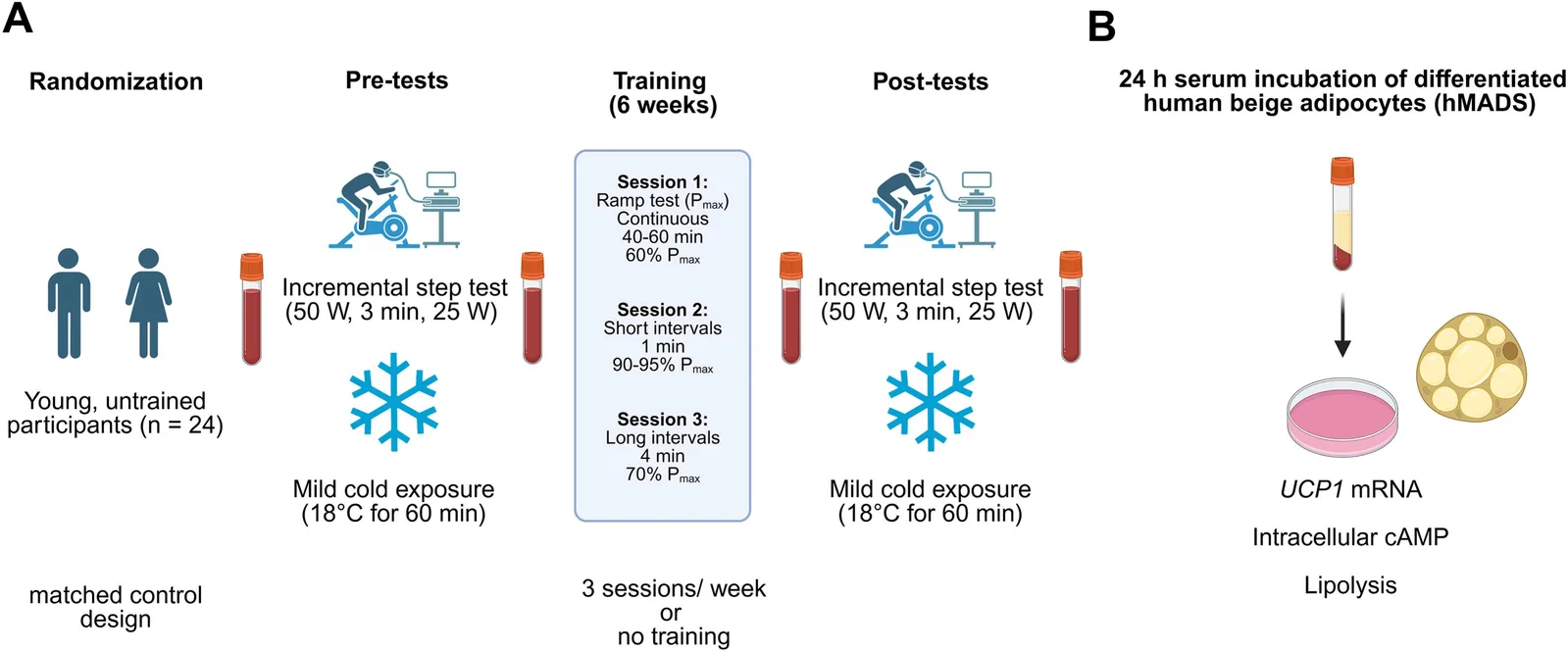
Overview of the experimental design of the study. A, Randomized matched control trial of endurance training intervention (*n* = 24) with pre-post testing including collection of resting and exercise−/cold-conditioned venous blood. B, Cell culture experiments using individual resting and exercise−/cold-conditioned serum from participants of the training group as a treatment.

Participants were randomly allocated to either a six-week endurance training intervention or a matched control group. Physiological responses to mild cold exposure and incremental step test performance performance were assessed before and after the intervention. In addition, serum collected at rest and following exercise or cold exposure was used for complementary cellular experiments in differentiated human beige adipocytes (Fig. 1).

#### Exercise intervention and training

All 12 participants of the training group trained for 3 days/week over 6 weeks with at least 1 day of rest between training days. Exercise training and all tests were performed at the Prevention Center of the Technical University of Munich. To increase motivation and effort during training, participants were supervised and trained in groups of up to 6 people during each of the sessions. The training protocol consisted of a weekly ramp test until maximal voluntary exhaustion (60 W, 20 W/min) early in the week (training day 1) to assess weekly individual maximal power (P_max_) on the cycle ergometer. After a 10 min active recovery (10 min at 60 W) this test was followed by 40–60 min of continuous cycling at 60% P_max_. The weekly reassessment of P_max_ allowed training workloads to be adjusted throughout the intervention, thereby maintaining the prescribed relative exercise intensity as participants' fitness improved. On training day 2 and day 3 participants performed short intervals (90–95%, 8–13 × 1 min with 1 min rest at 50%) and long intervals (70%, 4–16 × 4 min with 2 min rest at 50%), respectively. Each session started with a warm-up and ended with a cool-down. All 12 participants in the control group were instructed to neither change their physical activity level nor their habitual diet. To ensure adherence to the instructions, participants in the control group filled out weekly physical activity logs.

#### Incremental step test

Before and after the exercise intervention all participants performed cardiopulmonary exercise testing (CPET) through an incremental step test (50 W, 25 W, 3 min) until maximal voluntary exhaustion on a cycle ergometer (Lode Excalibur Sport, Lode, Groningen, Netherlands). Respiratory gases were continuously measured with the Cortex Metalyzer 3B (Cortex, Leipzig, Germany) and monitored with Meta Soft Studio (Cortex, Leipzig, Germany). Further, heart rate and ECG were measured with a 12-channel system (Custo Cardio 300, Custo Med, Bayern, Germany). Blood lactate levels were measured from capillary blood on a lactate analyzer (EKF Diagnostic; Biosen S-line). We ensured that the participants had a minimum of 3 days of rest between the last training session and the post-test.

#### Cold exposure challenge

Before (pre-test) and after (post-test) the exercise intervention all participants were challenged by a mild cold exposure test (60 min, 18 °C). Respiratory gases were analyzed and measured at baseline in the morning after an overnight fast (10−12 h) and immediately after the cold exposure across 15 min in a supine position using the Cosmed QNRG (Cosmed, Rome, Italy). The clothing during the cold exposure experiment was standardized and each participant had to wear a shirt, shorts and socks. Respiratory measurements were performed in a semi-dark and quiet room with one supervisor present to prevent participants falling asleep. Oxygen consumption (V’O_2_), carbon dioxide production (V’CO_2_) and the resulting respiratory exchange ratio (RER) were assessed across each 15-min measurement. Furthermore, V’CO_2_ and V’O_2_ (both ml/min/kg) were normalized to the participants' individual body weight (kg) and the respective 15-min area under the curve (AUC) was calculated to enable statistical comparison of the metabolic responses to cold exposure. Skin temperature was measured during the cold challenge using 8 wireless thermosensors (iButtons DS-1921H Thermochron, Texas, USA) that were attached to the skin with adhesive tape. Eight sensors were placed standardized skin sites of each individual according to ISO 9886 [31] to assess overall mean skin temperature. Two cooling blankets (Maxi-Therm Lite, Gentherm Medical, Michigan, USA) were perfused with 18 °C cold water by a cold circulation thermostat (WCR-8, Witeg, Baden-Württemberg, Germany) to achieve a 360° cooling of the participants in a supine position. To assess excessive muscle shivering, blood lactate levels were measured from capillary blood each 15 min during the 60 min cold challenge.

#### Anthropometric measurements

Before and after the exercise intervention, on the day of the exercise tests, we assessed the following anthropometric measurements: body height (Stadiometer 213, Seca, Hamburg, Germany), body weight and body composition via bio-impedance analysis (InBody 770, InBody, Hessen, Germany) after an overnight fast (10–12 h) and a standardized water intake of 500 ml.

### Blood drawing and processing

Before and after the incremental step tests and mild cold exposures venous blood was collected in a 9 ml *Z*-gel and a 9 ml EDTA monovette (Sarstedt, Nordrhein-Westfalen) The collected blood was gently inverted four times before further processing. For serum processing, blood was left for 30 min at room temperature to allow clotting and then centrifuged (2.500 G, 4 °C, 10 min). Blood collected to prepare plasma was centrifuged immediately (2.500 G, 4 °C, 10 min). Blood serum and plasma were aliquoted in cryovials (Sarstedt, Nordrhein-Westfalen) on dry ice and stored at −80 °C. Plasma aliquots were transferred on dry ice to the Helmholtz Center Munich for the measurement of noradrenaline and adrenaline. Samples were labeled anonymously and tested in random order. For blood lactate concentrations during exercise tests and cold challenges, capillary blood was sampled from the earlobe using safety lancets and capillaries of a volume of 20 μl in hemolysis buffer.

### Noradrenaline analysis

Plasma catecholamines were quantified using high-performance liquid chromatography (HPLC) coupled with electrochemical detection (ECD), as previously described [32]. Sample preparation followed the protocol provided by RECIPE Chemicals + Instruments GmbH for catecholamine extraction in human plasma with the ClinRep® complete kit, which includes all necessary reagents and materials for the extraction of the desired analytes. Due to the limited volume of human plasma available, modifications to the standard protocol were implemented. Specifically, 500 μL of human plasma was mixed with 10 μL of internal standard (DHBA, 0.25 ng/μL). The resulting solution was charged to the solid phase extraction column, which was then shaken for 15 min. Solvent was removed using a vacuum manifold, and the column was washed three times with 1 mL of wash solution to eliminate interfering substances. After drying the column, 100 μL of elution reagent was added. The column was shaken for 15 min and then the catecholamines were eluted from the extraction column via centrifugation. A 24 μL aliquot of the final eluate was injected into the HPLC-ECD system for analysis. Minor adjustments were also made to the detection parameters compared to our previous publication. The detector potential was set to 0.75 V against an Ag/AgCl reference electrode. The flow rate was adjusted to 0.9 mL/min, and the column temperature was maintained at 40 °C. With these changes, the retention times of the desired analytes were as follows: noradrenaline at 2.9 min, adrenaline at 3.2 min, the internal standard at 4.1 min, and dopamine at 5.8 min.

### Cell culture of human multipotent adipose-derived stem cells

**Fig. S1:**
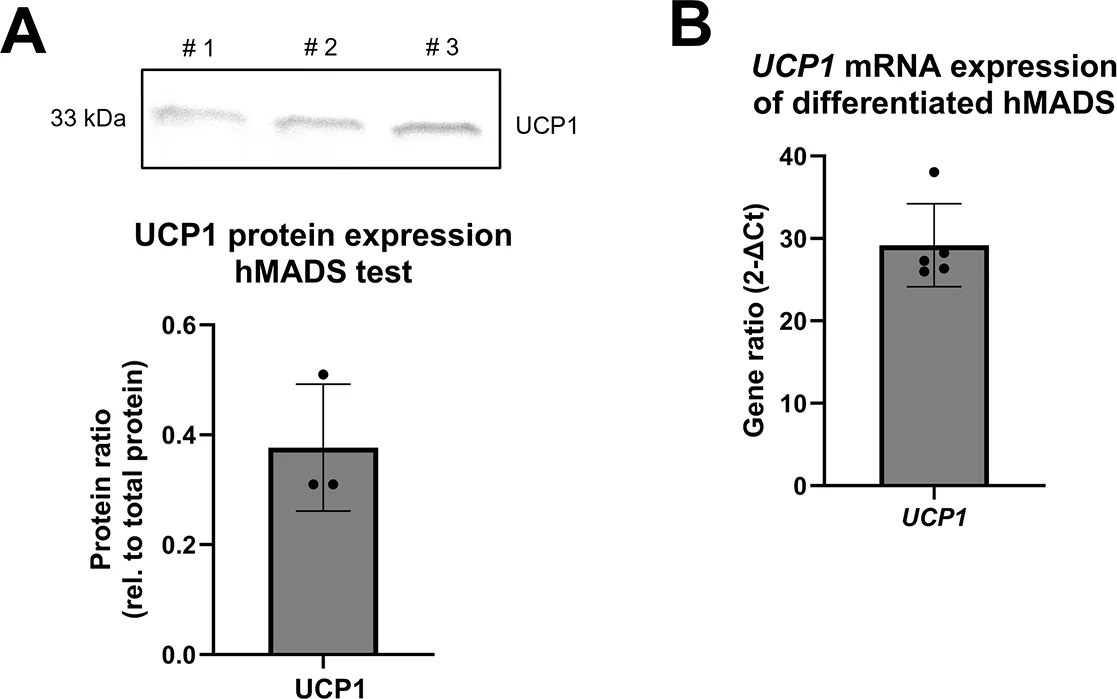
Expression of UCP1/*UCP1* levels in differentiated human beige adipocytes from hMADS cells on day 14 of differentiation. A, UCP1 protein levels relative to total protein content (*n* = 3), which was assessed by 0,25% coomassie staining of PVDF membrane. B, *UCP1* mRNA levels quantified via qPCR (*n* = 5). Data are presented as mean ± SD (A-B). Each dot represents one biological replicate.

Human multipotent adipose-derived stem (hMADS) cells from Christian Dani's lab were obtained and differentiated respectively [33]. Cells were cultivated at passage 14 and plated at passage 16 for experiments. Cells were grown using growth media (DMEM low glucose, 10% FBS, 1% Penicillin-Streptomycin, 1% HEPES). Once 100% confluency was reached, differentiation was induced (Day 0) by using induction media (DMEM low glucose, HAM's F12, 10% FBS, 1% Penicillin-Streptomycin, 1% HEPES, 5 μg/ml Insulin, 10 μg/ml Transferrin, 0.2 nM T3, 1 μM Rosiglitazone, 100 μM IBMX, 1 μM Dexamethasone, 200 μM 8-Br-cAMP). After 72 h (Day 3), induction media was replaced with differentiation media (DMEM low glucose, HAM's F12, 10% FBS, 1% Penicillin-Streptomycin, 1% HEPES, 5 μg/ml Insulin, 10 μg/ml Transferrin, 0.2 nM T3, 1 μM Rosiglitazone). Differentiation media was changed every other day until differentiation was finished on day 12. Expression of UCP1 levels in the differentiated beige adipocytes was verified via qPCR and Western Blot analysis (Fig. S1).

#### Serum incubation

Differentiated adipocytes were treated in triplicates with 10% resting, exercise- or cold-conditioned human serum of each participant. The untreated control condition for all experiments was treated with 10% FBS (Fetal Bovine Serum, PAN-Biotech, Bayern) only.

#### RNA extraction from cell culture samples

After overnight incubation (37 °C, 5% CO_2_) cells were washed with 250 μl PBS, lysed with 250 μl phenol/guanidine-based lysis reagent (QUIAzol, Quiagen, Nordrhein-Westfalen, Germany) and frozen on dry ice. On the next day, samples were thawed, vortexed and sat at room temperature for two minutes. Then 150 μl chloroform (Roth, Baden Württemberg, Germany) was added to each sample and samples were centrifuged at (12.000 rpm, 4 °C, 15 min). The aqueous phase containing the RNA was transferred in a fresh labeled tube on ice and 125 μl isopropanol (Merck, Hessen, Germany) as well as 1 μl glycogen (Merck, Hessen, Germany) was added for RNA precipitation. After a quick vortex, samples were incubated at −20 °C for 15 min. After precipitation, samples were vortexed and sat at room temperature for 10 min. After centrifugation (12.000 rpm, 4 °C, 10 min), the supernatant was discarded with a micro pipet. RNA pellet was resuspended in 250 μl cold 80% pure EtOH (Merck, Hessen, Germany), vortexed and centrifuged (12.000 rpm, 4 °C, 10 min). After a second washing step the RNA pellet was air dried for 30 min and eluted in 18 μl pure water (Roth, Baden Württemberg, Germany). RNA was quantified by fluorescence using the Qubit (Thermo Fisher Scientific, Hessen, Germany). RNA purity was controlled by the absorbance ratios 260/280 and 260/230 of the NanoDrop (Peqlab, Bayern, Germany).

#### mRNA expression analysis by RT-qPCR

1 μg RNA per replicate was used to synthesize cDNA (RevertAid First Strand cDNA Synthesis Kit, Thermo Fisher Scientific, Hessen, Germany). qPCR was performed using SYBR green-dye (SYBR Green Master Mix, Thermo Fisher Scientific, Hessen, Germany) to quantify mRNA expression of human *UCP1* (Forward: 5’-ACAGCACCTAGTTTAGGAAG-3′; Reverse: 5’-CTGTACGCATTATAAGTCCC-3′), human *TBP* (Forward: 5’-GCCAGAGTGAAGAACAG-3′; Reverse: 5’-GAAGTCCAAGAACTTAGCTG-3′) and human *HPRT1* (Forward: 5’-CCTGGCGTCGTGATTAGTGAT-3′; Reverse: 5’-AGACGTTCAGTCCTGTCCATAA-3′) using primer pairs (KiCqStart-Primer, Merck, Hessen, Germany) at a final concentration of 100 nM.

#### Western blot analysis

Rabbit polyclonal UCP1 antibody (ab23841, Abcam, Cambridge, UK) was diluted 1:2000 in TBST supplemented with 5% BSA (Bovine Serum Albumin Fraction V, Merck, Hessen, Germany). Anti-mouse HRP-linked antibody (07076S, Cell Signaling, Leiden, Netherlands) was diluted 1:2000. UCP1 protein expression was normalized to total protein for each sample using 0.1% Coomassie stain.

#### Lipolysis assay

Isoproterenol-stimulated lipolysis in cultured adipocytes was assessed by quantifying glycerol release using a commercial colorimetric assay (Lipolysis Assay, Akrivis Bio, MA-0189; assay range 0–10 nmol). Lipolysis was induced by treating cells in 96-well plates with 100 nM isoproterenol in the presence of 500 μM 3-isobutyl-1-methylxanthine (IBMX), a phosphodiesterase inhibitor, for 30 min. Following stimulation, 150 μL of culture supernatant was collected from each well, and free glycerol concentration was determined according to the manufacturer's instructions. Cells were lysed per well and calculated for total protein concentration (see following chapter 2.7.6.). Finally, glycerol release was expressed as nmol glycerol per mg protein.

#### Intracellular cAMP assay

Following lipolysis stimulation (Section 0), the same cultured adipocytes were washed with DPBS and lysed in ice-cold 0.1 M HCl supplemented with 500 μM IBMX to inhibit phosphodiesterase activity. Cells were incubated on ice for 5 min with gentle rocking before the remaining cell material was scraped from the wells. Cell lysates were centrifuged (10.000 G, 4 °C, 10 min,) to pellet cellular debris. The resulting supernatants were collected and total protein concentrations were determined using a Qubit 4 Fluorometer (Thermo Fisher Scientific, Hessen, Germany). Intracellular cAMP concentrations were subsequently quantified using a commercial cAMP Assay Kit (Abcam, ab290713) in the acetylated assay format according to the manufacturer's instructions to enhance assay sensitivity (assay range: 0.078–20 pmol/mL; intra-assay CV: 4–6%; inter-assay CV: 13–19%).

### Statistical analysis

Statistical analyses were performed using GraphPad Prism Version 10.6.1 (GraphPad Software, Boston, MA). Normality of data sets was assessed via QQ-plots. Values are expressed as means ± standard deviation (SD) or standard error of the mean (SEM) as stated in the figure legend. Statistical significance was set at *p* < 0.05.

To evaluate if the training group responded differently over time due to the endurance training intervention, two-way repeated measures ANOVA or a mixed-effects model in case of missing data points within a data set were used. Factors ‘Group’ (Training versus Control) and ‘Time’ (Pre-test versus Post-test) were included to test for ‘Group x Time’ interaction and respective mean effect sizes (ES) with confidence intervals (CI) were calculated.

Separate repeated measures two-way ANOVA with multiple comparisons was used to analyze the acute response to cold exposure such as the 15-min AUC assessments of oxygen consumption and carbon dioxide production before and after mild cold exposure, the increase in EE in response to mild cold exposure and *UCP1* mRNA levels (∆Ct) of differentiated beige adipocytes treated with serum from before and after the training intervention.

Ordinary one-way ANOVA with multiple comparisons was used to analyze any differences in lipolysis and intracellular cAMP among the treatment conditions.

Multiple paired t-tests were calculated to assess statistical significance of the heart rate and blood lactate concentrations during the steps of the incremental step tests. Two-tailed t-test was used to compare skin temperature change during mild cold exposure between groups.

## Results

### Randomized matched control trial increased maximal aerobic capacity in response to endurance training

**Fig. 2:**
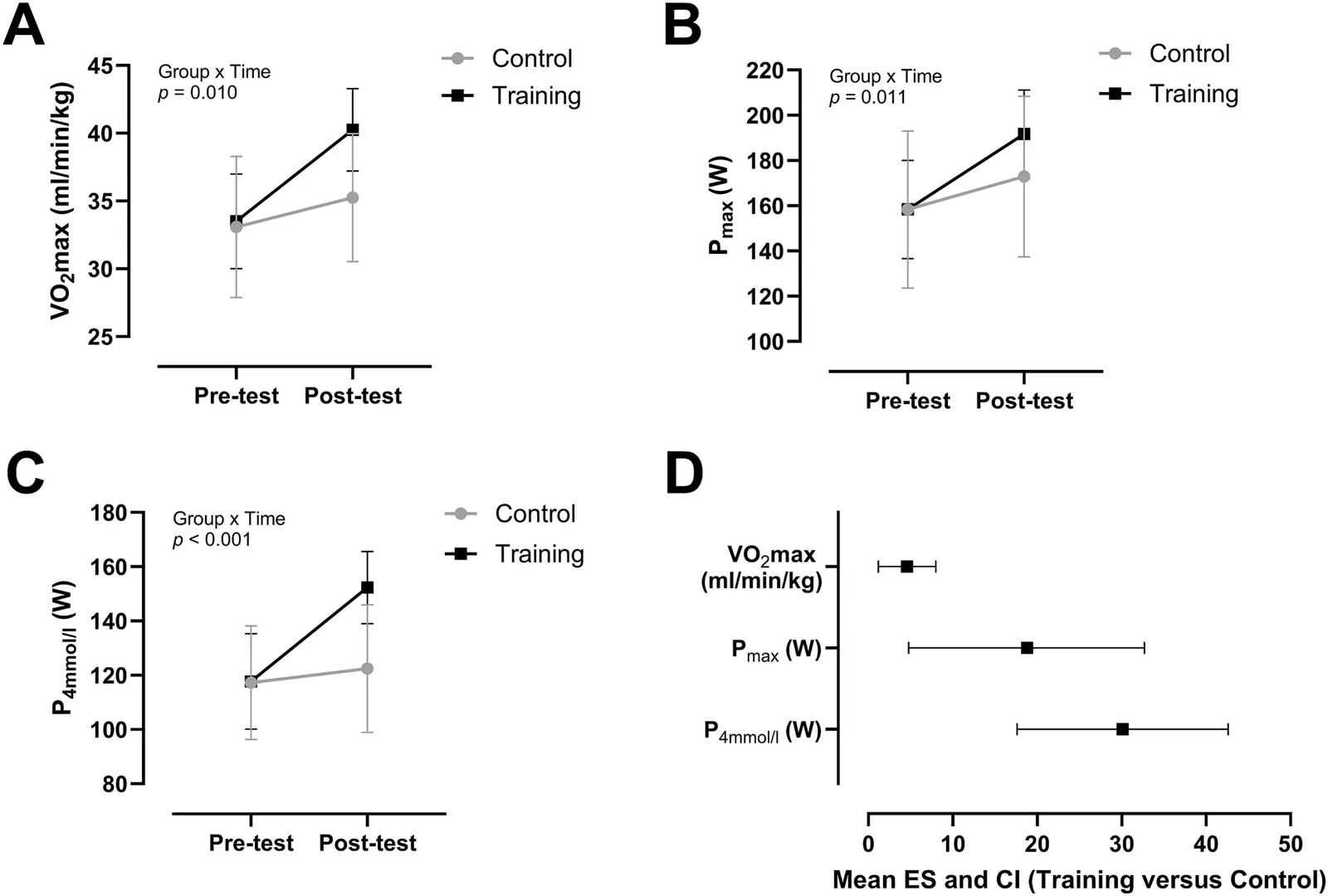
Effect of six weeks of fartlek-style endurance training on the V˙O_2_max (A), P_max_ (B) and P_4mmol/l_ (C) in control and training group including mean ES and 95% CI of measured mean differences (D). Data are presented as mean ± SD (A-C) and CI (D). Statistical significance was determined by two-way repeated measures ANOVA or mixed effects model. Control (*n* = 12) and Training (*n* = 12).

**Fig. S2:**
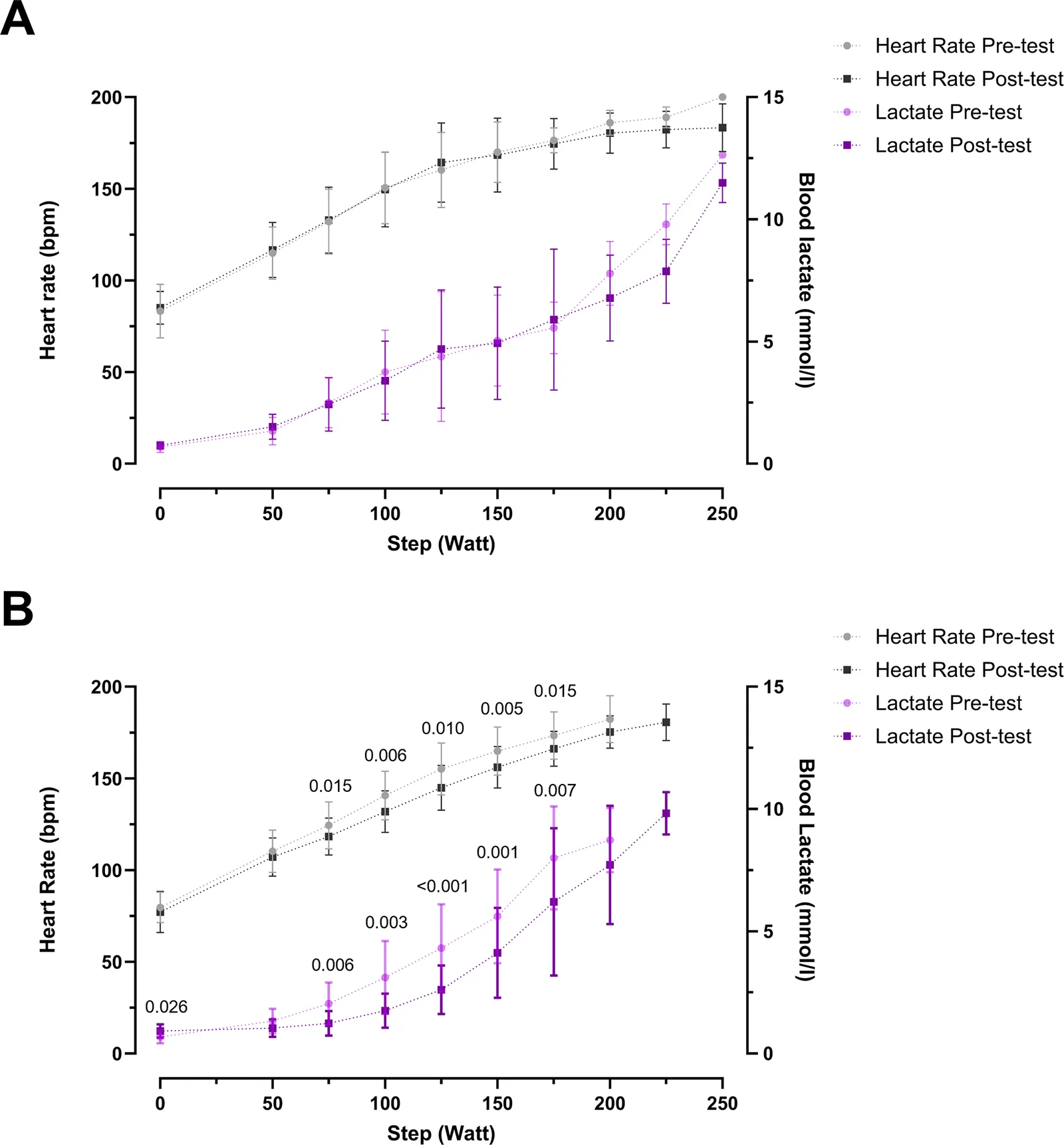
Heart rate (bpm) and blood lactate concentration (mmol/l) during the step tests until maximal voluntary exhaustion of the control group (A) and training group (B). Step test performance was assessed during cardiopulmonary exercise testing (CPET) during pre-test and post-test. Statistical significance was determined by paired ttest and *p*-values are indicated in the figures. Data are presented as mean ± SD (A-B). Control (*n* = 12) and Training (*n* = 12).

To assess the effects of endurance training on oxygen uptake and energy expenditure, twelve healthy young participants completed six weeks of fartlek-style endurance training three times per week, with individual intervention periods occurring between February and June 2026.Twelve sex-matched participants served as controls to account for effects due to increasing outside temperatures, which are associated with decreased BAT activity as judged by ^18^F-FDG PET scans [7]. The training group increased their VO_2_max from 34 ± 6 ml/min/kg to 40 ± 4 ml/min/kg (*p* < 0.001), P_max_ from 158 ± 34 W to 192 ± 31 W (*p* < 0.001) and P_4mmol_ from 118 ± 25 W to 152 ± 20 W (*p* < 0.001). Whereas no substantial change was observed in VO_2_max (33 ± 8 ml/min/kg to 35 ± 7 ml/min/kg) and P_4mmol/l_ (117 ± 33 W to 122 ± 37 W) in the control group but for P_max_ from 158 ± 55 W to 173 ± 56 W (*p* = 0.012). A significant ‘Group x Time’ interaction was detected for V˙O_2_max (*F*(1,22) = 7.75, *p* = 0.011, η^2^p = 0.346) (Fig. 2A), P_max_ (*F*(1, 22) = 7.75, *p* = 0.011, η^2^p = 0.346) (Fig. 2B) and P_4mmol/l_ (*F*(1,21) = 25.10, *p* < 0.001). (Fig. 2C). Post hoc analysis demonstrated a greater increase in the training group compared with controls for V˙O_2_max (Mean ES: 5 ml/min/kg, 95% CI: 1 to 8, *p* = 0.010), P_max_ (Mean ES: 19 W, 95% CI: 5 to 33, *p* = 0.011) and P_4mmol/l_ (Mean ES: 30 W, 95% CI: 18 to 43, *p* < 0.001) (Fig. 2D). Further, heart rate and blood lactate during each of the submaximal intensities of the step test significantly decreased (Fig. S2). Consequently, these results show that three sessions of 40–60 min of fartlek-style endurance training per week across six weeks suffice to induce strong cardiorespiratory and metabolic adaptations.

### Endurance training does not alter the metabolic response to mild cold exposure

**Fig. 3:**
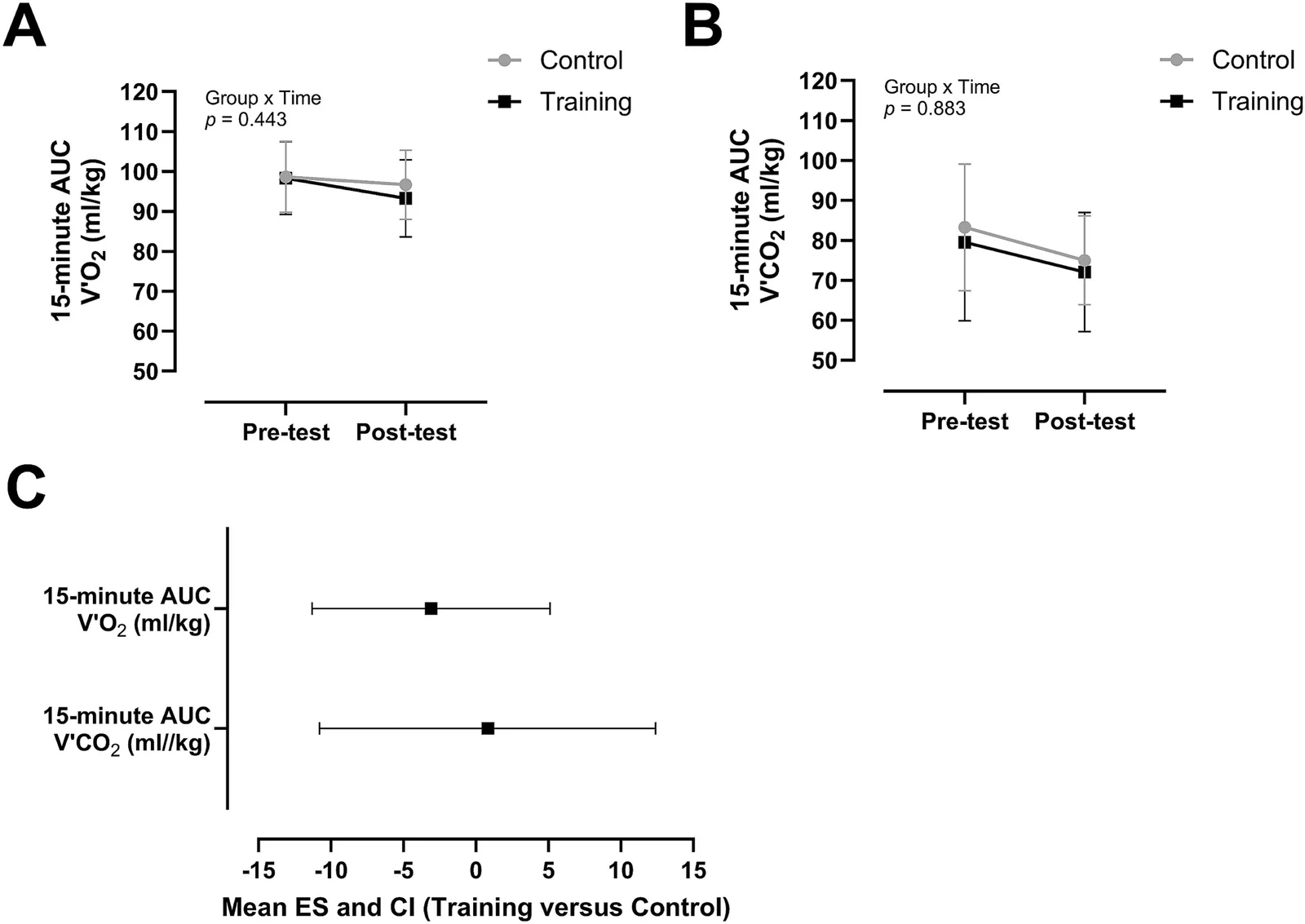
Response of 15-min AUC of oxygen uptake (V’O_2_) (A) and carbon dioxide production (V’CO_2_) (B) to mild cold exposure (18 °C, 60 min) before and after 6-weeks of endurance training including mean ES and 95% CI of measured mean differences (C). Data are presented as mean ± SD (A-B) and CI (C). Statistical significance was determined by two-way repeated measures ANOVA. Control (*n* = 12) and Training (*n* = 12).

**Fig. S3:**
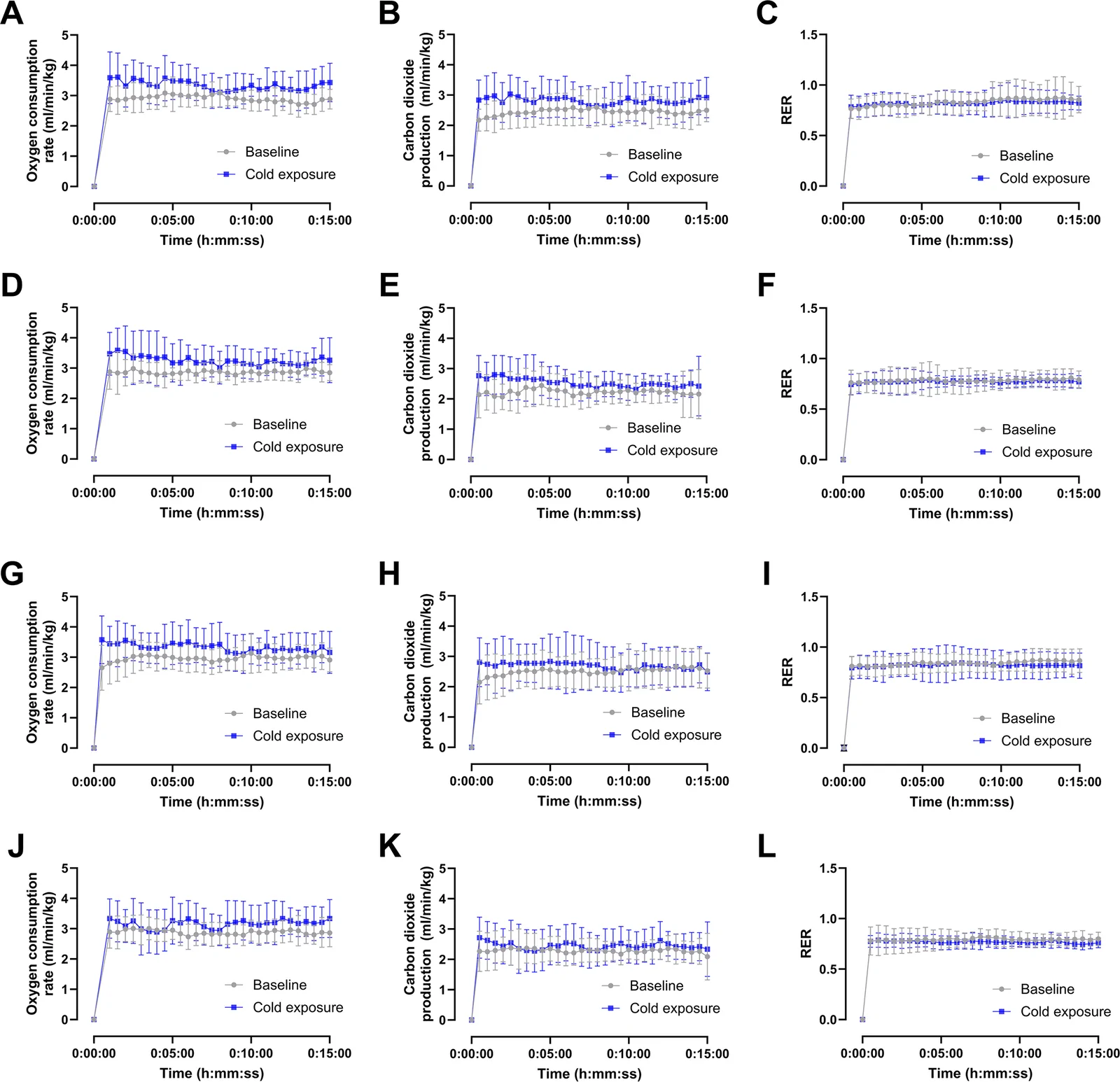
Oxygen consumption rate (ml/min/kg), carbon dioxide production (ml/min/kg and respiratory exchange ratio (RER) across the 15-min cold exposure experiments at baseline and after cold exposure. A-C, Pre-test of control group. D-F, Post-test of control group. G-I, Pre-test of training group. J-L, Post-test of training group. Data are presented as mean ± SD (A-B). Control (*n* = 12) and Training (*n* = 12).

To investigate whether six weeks of endurance training would alter the oxygen uptake response to cold exposure, the oxygen uptake (V’O_2_) and carbon dioxide production (V’CO_2_) were measured during 15-min indirect calorimetry assessments before (baseline) and after 60 min of mild cold exposure (18 °C) during both pre-test and post-test of the conducted endurance training intervention (Fig. 3A-C). The respirometry response to the cold challenge was normalized to body weight (ml/min/kg). Oxygen consumption and carbon dioxide production was increased compared to the baseline measurement after mild cold exposure in both control and training group without affecting the respiratory exchange ratio (RER) (Fig. S3). Each group's respirometry response to the cold exposure was also quantified by calculating the area under the curve (AUC) of each 15-min indirect calorimetry assessment (15-min AUC) in ml/kg. Across the six-week intervention the control group the 15-min AUC V’O_2_ from 99 ± 14 ml/kg to 97 ± 14 ml/kg (Fig. 3A) barely changed and the 15-min AUC V’CO_2_ decreased from 83 ± 16 ml/kg to 75 ± 11 ml/kg (Fig. 3B). The training group decreased the 15-min AUC V’O_2_ from 98 ± 14 ml/kg to 93 ± 15 ml/kg (Fig. 3A) and the 15-min AUC V’CO_2_ from 90 ± 20 ml/kg to 72 ± 15 ml/kg (Fig. 3B). These changes showed no ‘Group x Time’ interactions. Post hoc analysis showed that the training group compared to controls slightly reduced 15-min V’O_2_ (Mean ES: −3 ml/kg, 95% CI: −11 to 5, *p* = 0.443) and barely increased V’CO_2_ (Mean ES: 1 ml/kg, 95% CI: −11 to 12, *p* = 0.883) (Fig. 3C).

**Fig. 4:**
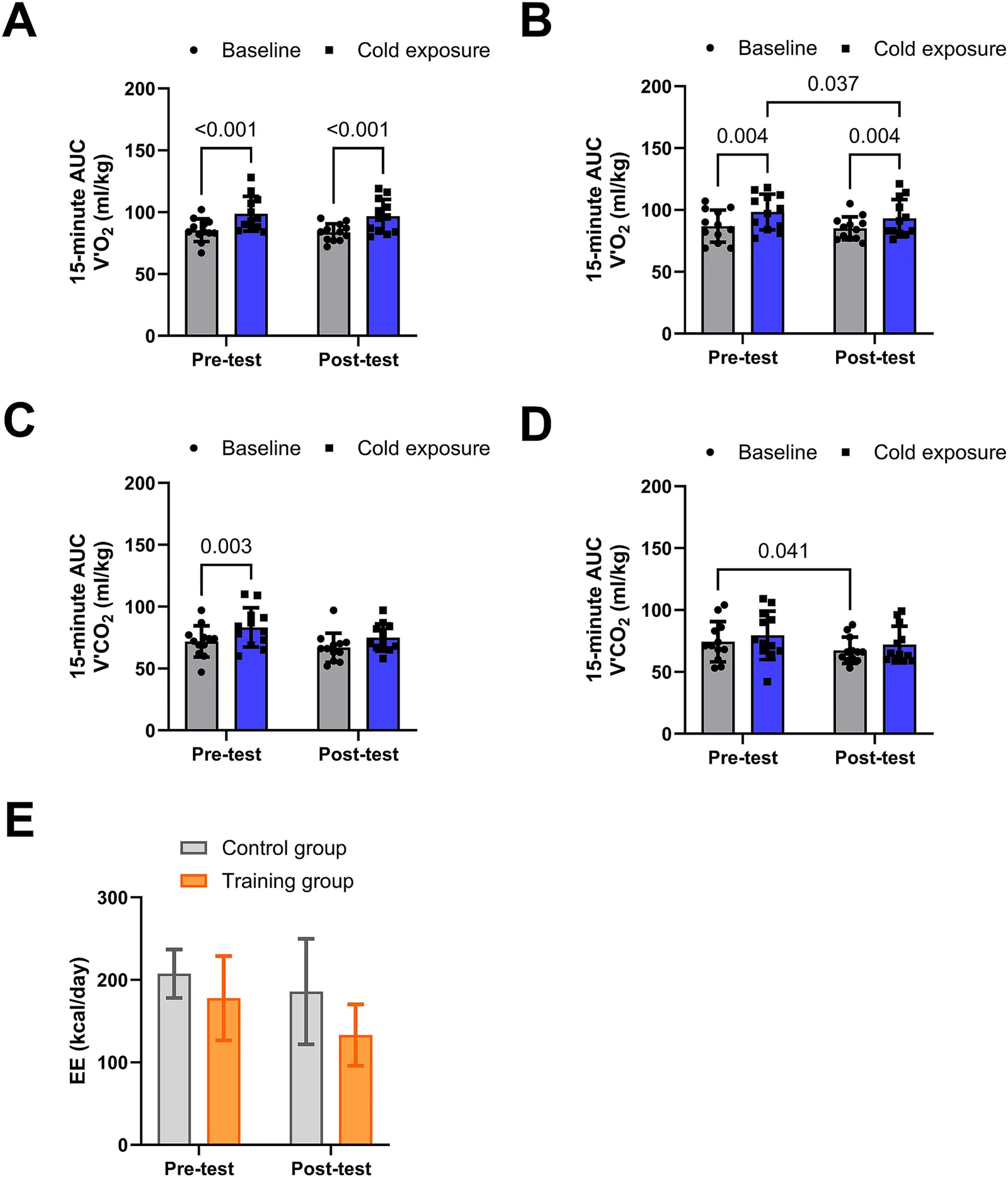
Acute responses of 15-min V’O_2_ (A-B), 15-min V’CO_2_ (C-D) and daily EE (E) to mild cold exposure (18 °C, 60 min) before and after 6-weeks of endurance training. A, control group's 15-min V’O_2_ (ml/kg). B, Training group's 15-min V’O_2_ (ml/kg). C, Control group's 15-min V’CO_2_ (ml/kg). D, Training group's 15-min V’CO_2_ (ml/kg). E, Control and Training group's cold-induced daily energy expenditure (EE) due to the cold response before and after the exercise intervention. Statistical significance was determined by two-way repeated measures ANOVA including multiple comparison and *p*-values are indicated in the figures. Data are presented as mean ± SD. Control (*n* = 12) and Training (*n* = 12). Each dot (A-D) represents one human.

Acutely within conditions, cold-induced 15-min AUC of V’O_2_ was significantly increased after cold exposure in the control group from 86 ± 9 to 99 ± 14 ml/min/kg (*p* < 0.001) during the pre-test as well as after 6 weeks from 85 ± 7 to 97 ± 14 ml/min/kg (*p* < 0.001) during the post-test (Fig. 4A). Likewise, the training group experienced an acute cold-induced increase in 15-min AUC of V’O_2_ from 88 ± 12 to 98 ± 14 ml/min/kg (*p* = 0.004) during pre-test and from 85 ± 9 to 93 ± 15 ml/min/kg (*p* = 0.004) during post-test (Fig. 4B). Within the training group, the acute cold-induced increase in 15-min AUC V'O₂ was smaller at post-test than at pre-test (*p* = 0.037).

In contrast, the acute response to mild cold exposure of the 15-min AUC of V’CO_2_ was only significantly increased after cold exposure in the control group from 72 ± 13 to 83 ± 16 ml/kg (*p* = 0.003) during the pre-test but not after 6 weeks from 67 ± 12 to 75 ± 11 ml/kg during the post-test (Fig. 4B). Also, the training group showed no significant acute cold-induced increases in 15-min AUC of V’CO_2_ from 74 ± 17 to 79 ± 20 ml/kg during pre-test and from 67 ± 11 to 72 ± 15 ml/kg during post-test (Fig. 4B).

**Fig. S4:**
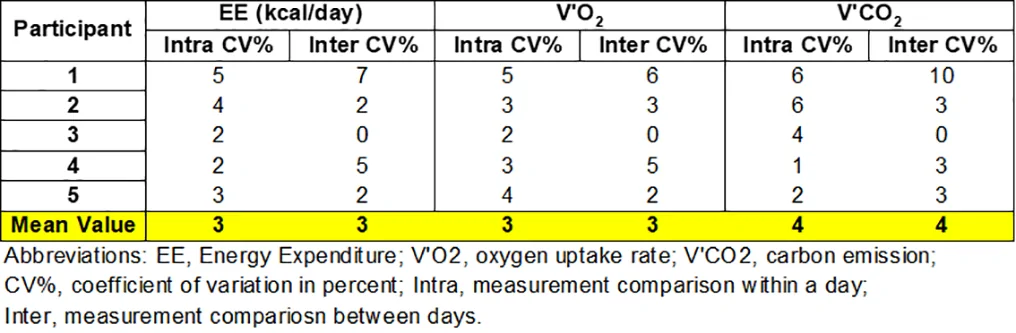
Calculation of the mean coefficient of variation in percent (CV%) of our respirometry device when performing multiple measurements with the same participants under standardized conditions. Comparison of energy expenditure (EE), oxygen consumption rate (V’O_2_) and carbon dioxide production (V’CO_2_) of 5 participants within two 15 min indirect calorimetry measurements on the same day (intra CV%) and on two consecutive days (inter CV%).

Overall, this reflects a 5% difference in the cold-induced oxygen consumption in the training group after training. The measured effect is above the coefficient of variation (CV) of our respirometry device (CV = 3%). CV of the respirometry device was assessed via inter-intra-individual comparison testing (Fig. S4) performed on separate days under similar conditions.

Thus, the acute cold-induced response of oxygen consumption was attenuated during the post-test but did not show a significant ‘Group x Time’ interaction. Cold-induced thermogenesis was quantified by daily individual EE (kcal/day) in both Control group (pre-test: 208 ± 101 kcal/day; post-test: 186 ± 221 kcal/day) and Training group (pre-test: 178 ± 177 kcal/day; post-test: 133 ± 129 kcal/day) (Fig. 4E). The cold-induced EE was not significantly altered in the training group compared to the control group (48 ± 64 kcal/day versus 22 ± 64 kcal/day respectively) (Fig. 4E).

### Time decreases the supraclavicular skin temperature during mild cold exposure

**Fig. 5:**
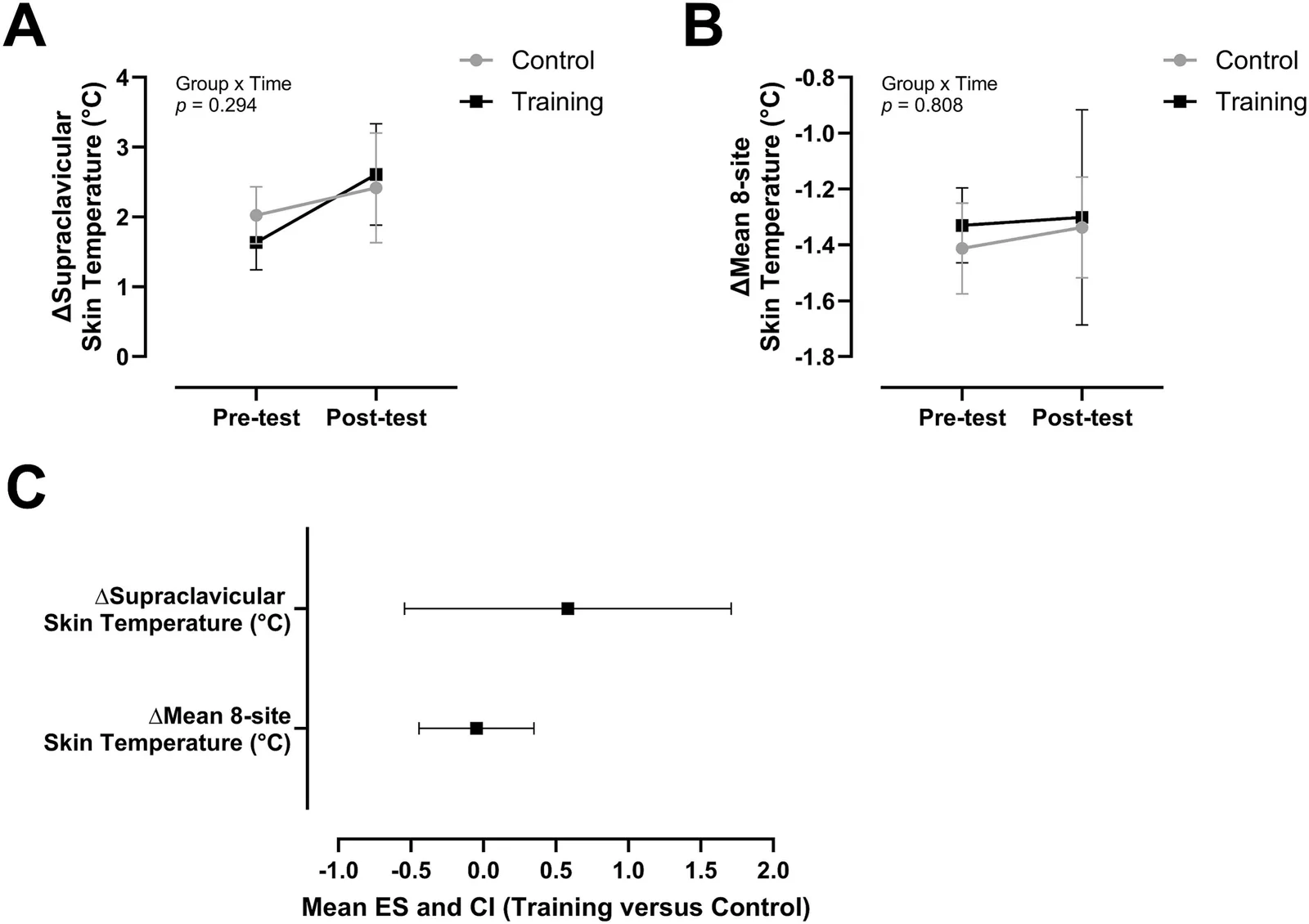
Effect of six weeks of fartlek-style endurance training on ∆supraclavicular skin temperature (A), ∆mean 8-site skin temperature (B) including mean ES and 95% CI of measured mean differences (C). Data are presented as mean ± SD (A-B) and CI (C). Statistical significance was determined by two-way repeated measures ANOVA. Control (*n* = 12) and Training (*n* = 12).

In humans, brown adipose tissue is located especially in the supraclavicular area of the upper body [34]. To estimate heat production by this BAT depot, we placed a thermosensor on the skin above the supraclavicular region as a surrogate marker while also calculating the mean 8-site skin temperature as described elsewhere [35]. No significant ‘Group x Time’ interaction was detected for the supraclavicular skin temperature change during cold exposure (*F*(1, 22) = 1.15, *p* = 0.294, η^2^p = 0.050) (Fig. 5A). No significant ‘Group x Time’ interaction could be detected for the mean 8-site skin temperature change during cold exposure (*F*(1, 22) = 0.0604, *p* = 0.808, η^2^p = 0.003) (Fig. 5B). However, the change in supraclavicular skin temperature during the cold exposure revealed significance for the factor ‘Time’ (*F*(1, 22) = 6.34, *p* = 0.020, η^2^p = 0.223). Post hoc analysis demonstrated a higher but unsignificant increase of the supraclavicular skin temperature change during the 60-min cold exposure in the training group compared with controls for the supraclavicular skin temperature change (Mean ES: 0.6 °C, 95% CI: −0.5-1.7, *p* = 0.294) (Fig. 5C). In contrast, the mean 8-site skin temperature during cold exposure remained almost unaffected (Mean ES: -0.1 °C, 95% CI: −0.4-0.4, *p* = 0.808) (Fig. 5C).

**Fig. 6:**
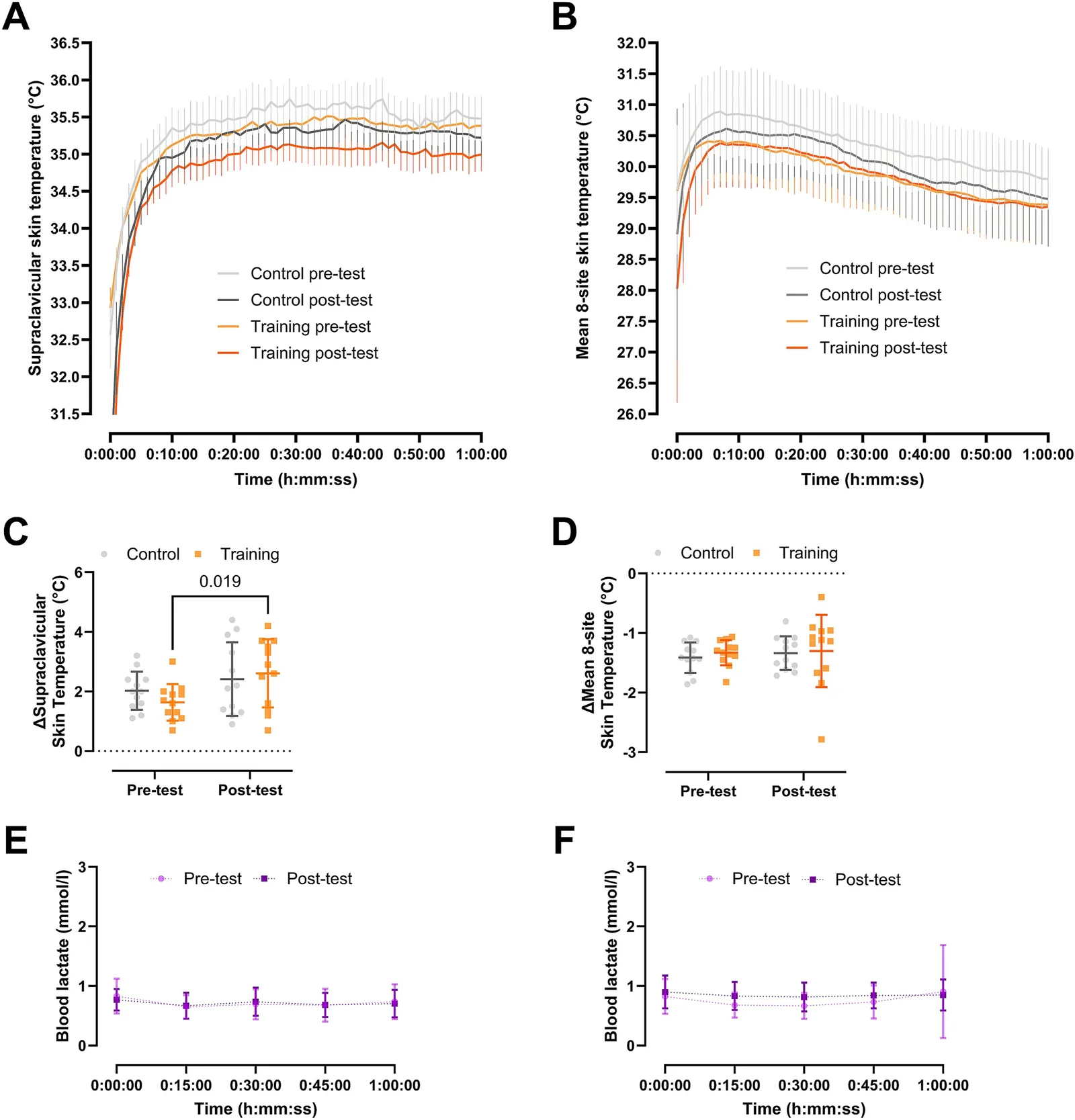
A, Supraclavicular skin temperature and B, mean 8-site skin temperature (°C) of both groups during 60-min mild cold exposure during pre- and post-test. C, Supraclavicular skin temperature change and D, mean 8-site skin temperature change (°C) of both groups during 60-min mild cold exposure during pre- and post-test. E, F, Blood lactate concentrations during the 60-min mild cold exposure before and after the exercise intervention of control and training group. Statistical significance was determined by two-way ANOVA and *p*-values are indicated in the figures. Data are presented as mean ± SD (C-F) and mean ± SEM (A-B). Each dot (*E*-F) represents one human. Control (*n* = 12) and Training (*n* = 12).

Overall, the monitored supraclavicular skin temperatures during 60-min mild cold exposure were significantly lower at post-tests than at pre-tests in both groups (Training group: −0.5 ± 0.1 °C; Control group: −0.3 ± 0.0 °C) (all *p* < 0.001) (Fig. 6A). The cold-induced supraclavicular skin temperature change was significantly higher during the post-test of the training group (Pre-test: 1.0 ± 0.4 °C; Post-test: 0.4 ± 0.4 °C; *p* = 0.019) (Fig. 6C).

To evaluate the efficacy of the cold exposure, we measured the mean 8-site skin temperature change during pre- and post-tests. Mean 8-site skin temperature decreased similarly in the training group by 1.3 ± 0.2 °C (pre-test) and 1.3 ± 0.6 °C (post-test) as well as in the control group by 1.4 ± 0.3 °C (pre-test) and 1.3 ± 0.3 °C (post-test) (Fig. 6B, D).

To monitor for possible excessive skeletal muscle shivering during the cold challenge, we measured blood lactate concentrations each 15th min (Fig. 6E,F) but found no significant differences in blood lactate levels.

### Exploratory analysis indicates an attenuated cold-induced plasma noradrenaline response following endurance training

**Fig. 7:**
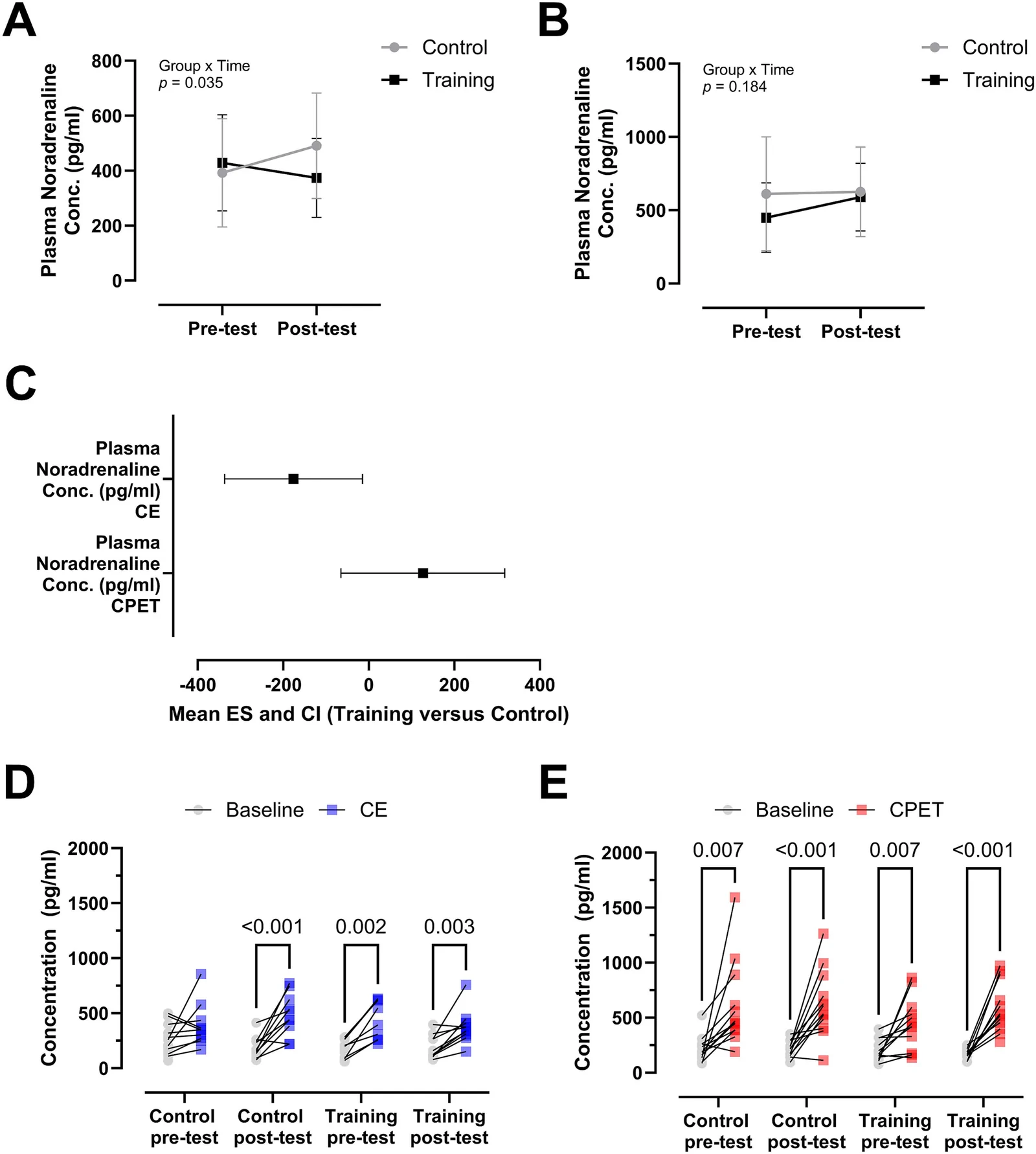
Plasma noradrenaline concentrations from venous blood collected before and after the six-week exercise intervention following 60-min mild cold exposure (CE) (A) and following cardiopulmonary exercise testing (CPET) until maximal exhaustion on a cycle ergometer (B) including mean ES and 95% CI of measured mean differences (C). Acute plasma noradrenaline response following CE (D) and CPET (E). Data are presented as mean ± SD (A-B) and CI (C). Statistical significance was determined by two-way repeated measures ANOVA or mixed effects model. Control (*n* = 9) and Training (*n* = 9).) Each dot (D-E) represents one human.

BAT thermogenesis in response to cold exposure is stimulated by noradrenaline which increases in human plasma after both mild cold exposure and a bout of exercise until maximal exhaustion (Fig. 7D,E). A significant ‘Group x Time’ interaction was detected for plasma noradrenaline levels in response to cold exposure (CE) (*F*(1, 16) = 5.33, *p* = 0.035) (Fig. 7A) but not for plasma noradrenaline concentration after cardiopulmonary exercise testing (CPET) (*F*(1, 22) = 1.88, *p* = 0.184, η^2^p = 0.077) (Fig. 7B). Post hoc analysis demonstrated a lower response in the training group compared with controls for plasma noradrenaline concentration in response to CE (Mean ES: −176 pg/ml, 95% CI: −337 to −14), *p* = 0.035) and higher but unsignificant plasma noradrenaline concentrations following CPET (Mean ES: 127 pg/ml, 95% CI: −65 to 318, *p* = 0.184) (Fig. 7C). In response to cold exposure, the control group increased plasma noradrenaline concentrations from 257 ± 142 pg/ml to 392 ± 197 pg/ml during the pre-test and from 173 ± 101 pg/ml to 491 ± 192 pg/ml (*p* < 0.001) during the post-test (Fig. 7D). In response to cold exposure, the training group increased plasma noradrenaline concentrations from 169 ± 82 pg/ml to 429 ± 175 pg/ml (*p* = 0.002) during the pre-test and from 195 ± 108 pg/ml to 374 ± 144 pg/ml (*p* = 0.003) during the post-test (Fig. 7D). In response to CPET the control group increased plasma noradrenaline concentrations from 216 ± 118 pg/ml to 612 ± 388 pg/ml (*p* = 0.007) and the training group from 201 ± 99 pg/ml to 450 ± 237 pg/ml (*p* = 0.007) during pre-tests (Fig. 7E). After 6 weeks during post-tests, the control group increased plasma noradrenaline concentrations in response to acute exercise from 215 ± 73 pg/ml to 626 ± 306 pg/ml (*p* < 0.001) and the training group from 169 ± 45 pg/ml to 590 ± 231 pg/ml (*p* < 0.001) (Fig. 7E).

### Conditioned serum of the training group does not increase *UCP1* gene expression of beige adipocytes after 24 h in vitro

**Fig. 8:**
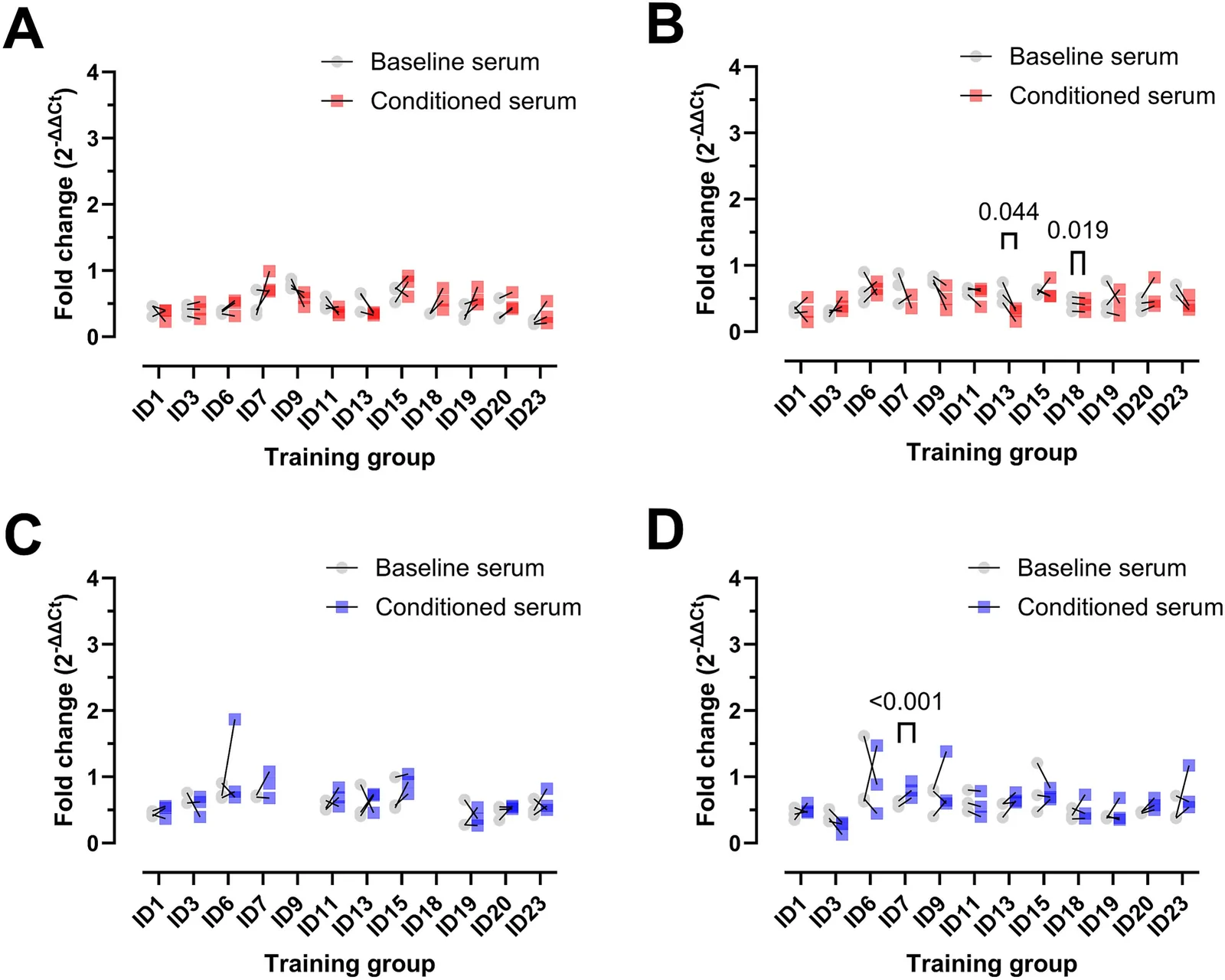
A-B, *UCP1* mRNA levels of human beige adipocytes treated with individual exercise-conditioned serum overnight for 24 h of pre-test (A) and post-test (B) in triplicates. C-D, *UCP1* mRNA levels of human beige adipocytes treated with individual cold-conditioned serum overnight for 24 h of pre-test (C) and post-test (D) in triplicates. Statistical significance was determined by two-way ANOVA (using ∆*Ct*) and *p*-values are indicated in the figures. Each dot (A-D) represents a cell culture replicate. Missing values are due to incomplete blood sampling.

UCP1-dependent thermogenesis in response to cold exposure is driven by adrenergic signaling such as via an increase in circulating noradrenaline. The thermogenic agonists noradrenaline was increased in human plasma after both mild cold exposure and a bout of exercise until maximal exhaustion (Fig. 7D-E). Thrice weekly endurance exercise for 6 weeks does lead to lower cold-induced plasma noradrenaline levels (Fig. 7A). However, the treatment of differentiated beige adipocytes with cold-/exercise conditioned serum from each individual participant for 24 h does not increase *UCP1* mRNA expression compared to individual resting serum (Fig. 8A-D). Few individual cold- and exercise-conditioned sera caused a significant increase (Fig. 8D) or decrease (Fig. 8B) of *UCP1* mRNA levels after 6 weeks of endurance training. Overall, relative *UCP1* gene expression of all serum treated adipocytes decreased compared to untreated beige adipocytes (Fig. 8A-D).

### Exercise-conditioned serum increases lipolytic responsiveness without increasing intracellular cAMP

**Fig. 9:**
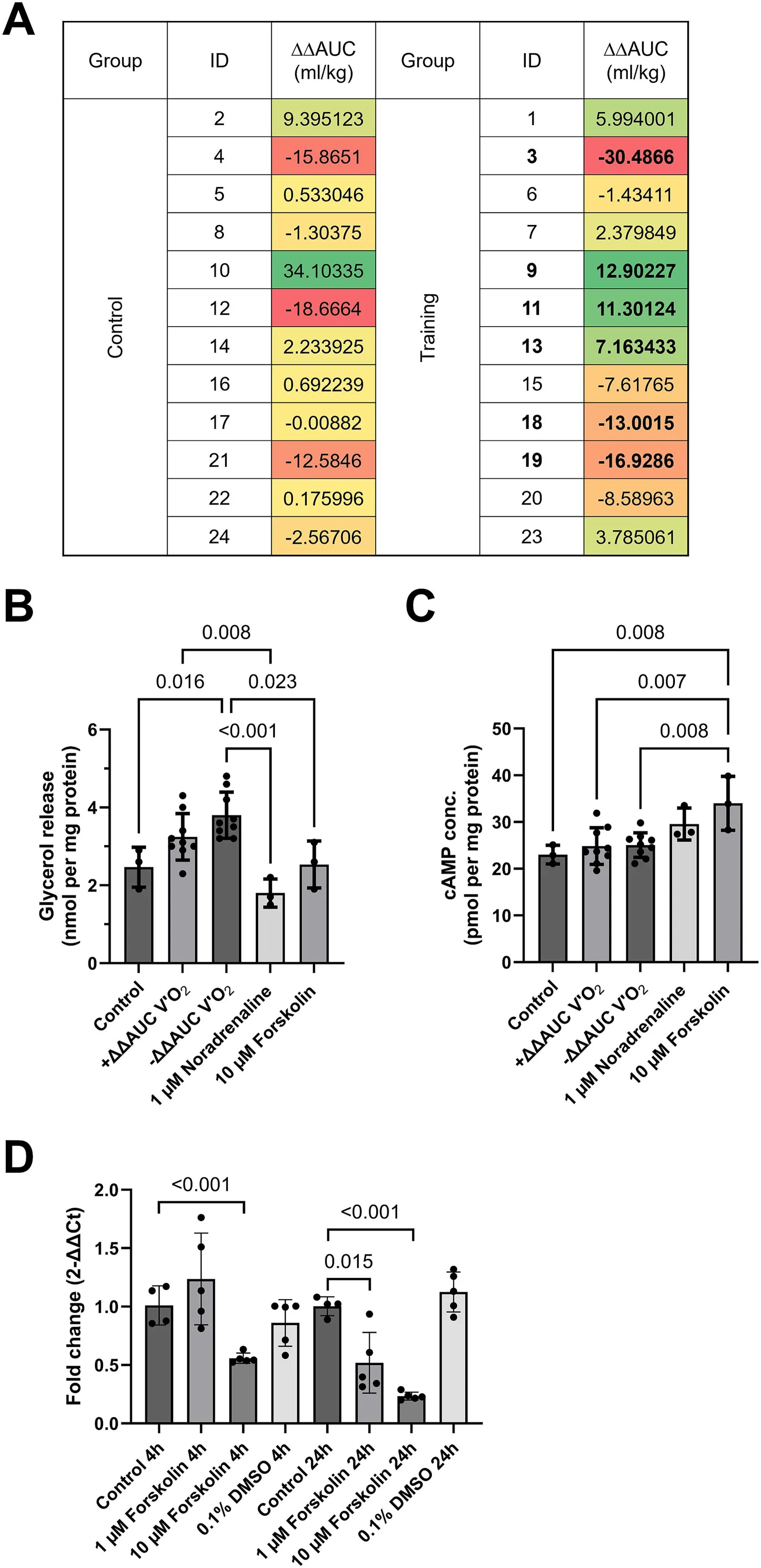
A, Schematic overview of differences in cold-induced 15-min AUC V’O_2_ (∆∆AUC) in ml/kg of participants of control and training group. Marked in bold are six participants of the training group that revealed an increase (+∆∆AUC) or decrease (−∆∆AUC) in their thermogenic response to cold exposure. B, Induction of lipolysis and C, intracellular cAMP levels via IBMX (500 μM) and isoproterenol (100 nM) for 30 min of differentiated human beige adipocytes that were treated with either serum from participants with a + ∆∆AUC, a -∆∆AUC, 1 μM noradrenaline or 10 μM forskolin in triplicates 24 h prior the experiment. D, *UCP1* mRNA expression levels of beige adipocytes treated with 1 μM and 10 μM forskolin and vehicle control (0.1% DMSO) after both 4 and 24 h with 5 replicates per condition. Statistical significance was determined by ordinary one-way ANOVA (using ∆Ct) and *p*-values are indicated in the figures (B-D). Data is presented as mean ± SD (B-D). Each dot represents a replicate (B-D).

The aim was to further characterize the functional response of beige adipocytes following serum treatment with serum collected during the post-tests from participants of the training group. The aim was to assess the thermogenic function of the beige adipocytes after serum treatment by quantifying catecholamine-induced (500 μM IBMX and 100 nM isoproterenol) lipolysis and intracellular cAMP levels since no change in *UCP1* mRNA could be detected after 24 h. Therefore, serum of 6 participants with the highest increase or decrease (+∆∆AUC: ID-9, ID-11 & ID-13; −∆∆AUC: ID-3, ID-18 & ID-19) in the cold-induced 15-min AUC V’O_2_ were chosen (Fig. 9A). -∆∆AUC sera significantly increased glycerol release from beige adipocytes by 1.3 ± 0.4 nmol per mg protein (*p* = 0.016) compared to control condition while treatment with thermogenic agonists noradrenaline (1 μM) or forskolin (10 μM) resulted in significantly lower glycerol release than treatment with -∆∆AUC sera (−2.0 ± 0.4 nmol per mg protein, *p* < 0.001; −1.3 ± 0.4 nmol per mg protein, *p* = 0.023) (Fig. 9B). Also, treatment with noradrenaline (1 μM) showed a lower glycerol release as treatment with -∆∆AUC sera (−1.4 ± 0.4 nmol per mg protein, *p* = 0.008) sera respectively (Fig. 9B). Additionally, intracellular cAMP levels were quantified to assess thermogenic activation in response to catecholamines after the 24 h treatments. Intracellular cAMP levels were significantly higher after treatment with 10 μM forskolin compared to untreated control (7 ± 3 nmol per mg protein, *p* = 0.008), −∆∆AUC (9 ± 2 nmol per mg protein, *p* = 0.008) and + ∆∆AUC (9 ± 2 nmol per mg protein, *p* = 0.007) (Fig. 9C). Within a final follow-up experiment the aim was to compare *UCP1* mRNA levels of beige adipocytes treated with a low (1 μM) or high (10 μM) dose of forskolin after both 4 and 24 h treatment (Fig. 9D). Treatment with 10 μM forskolin significantly reduced *UCP1* mRNA levels after 4 h (0.56 ± 0.04 fold change, *p* < 0.001) and 24 h (0.23 ± 0.03 fold change, *p* < 0.001) as well as 1 μM forskolin after 24 h (0.52 ± 0.26 fold change, *p* = 0.015). However, 1 μM forskolin resulted in a non-significant increase in *UCP1* mRNA levels after 4 h (1.24 ± 0.39 fold change).

## Discussion

In this randomized controlled trial, six weeks of supervised fartlek-style endurance training increased the V˙O_2_max on average by 6 ml/kg/min, maximal power output by 19 W and power at the 4 mmol/l lactate threshold by 30 W but did not increase physiological markers of cold-induced thermogenesis. Specifically, endurance training did not alter whole-body oxygen uptake or skin temperature during mild cold exposure. Moreover, exercise- or cold-conditioned serum failed to increase *UCP1* expression in cultured adipocytes. In contrast, the noradrenaline response to cold exposure was attenuated after training. Together, these findings suggest that short-term fartlek-style endurance training effectively increases cardiorespiratory and metabolic fitness but has little effect on the physiological response to mild cold exposure in healthy, young adults.

One distinctive feature of our study was the fartlek endurance intervention. Fartlek (“speed play”) training was developed in Sweden and is a hybrid of continuous endurance exercise with high intensity interval training (HIIT) without passive recovery periods [36]. The average increases of the V˙O₂max by 6 ml/kg/min and power at the 4 mmol/l lactate threshold by 30 W are at the upper end of those commonly reported for endurance training of a similar duration [37], [38], [journal], [40]. Although direct comparisons with other forms of endurance training require controlled studies, our findings suggest that cycle ergometer-based fartlek training may provide an effective and time-efficient approach for inducing cardiovascular and metabolic adaptations in exercise intervention studies.

Supraclavicular skin temperature decreased from pre- to post-test in both the training and control groups, suggesting a seasonal rather than an exercise training effect. This is consistent with repeated measurements showing lower cold-induced BAT activation during warmer months (May–September) in the same individuals [7]. The decrease in supraclavicular skin temperature in both groups may therefore reflect seasonal changes associated with the transition from winter to spring rather than an effect of exercise training.

Endurance training also reduced cold-induced plasma noradrenaline concentrations, whereas noradrenaline concentrations at rest and during exercise were unchanged. Previous studies have reported different findings. After 20 weeks of cycling (80% maximal heart rate, three sessions/week), plasma noradrenaline at the same absolute workload decreased by ∼50% (1371 ± 286 to 687 ± 64 pg/ml), but was unchanged at the same relative workload [41]. In contrast, a 10-week endurance training study reported higher plasma noradrenaline concentrations during 15-min treadmill running at 65–85% V˙O₂max after training [42], while an 8-week cycling intervention in older adults (64 ± 1.6 years) increased basal plasma noradrenaline by ∼24% [43].

Previous studies have proposed that skeletal muscle, rather than BAT, drives the metabolic benefits of the cold response. Recent findings suggest that muscle shivering, but not adipose tissue thermogenesis, increases with cold exposure [28]. If skeletal muscle shivering during the human mild cold response correlated to decreasing cooling temperatures, it might be possible that an increase of the maximal aerobic capacity affects the oxygen consumption during mild cold exposure. We assessed shivering indirectly by measuring capillary blood lactate during cold exposure but found no significant increases, consistent with previous cold-water immersion studies [44], [45]. Nevertheless, surface EMG would provide a more sensitive assessment of shivering than blood lactate.

We complemented the human study with cell culture experiments in which we treated differentiated human beige adipocytes for 24 h with resting, cold-conditioned, exercise-conditioned, or post-training serum. None of these treatments increased *UCP1* mRNA expression compared with resting serum. Most studies using hMADS-derived beige adipocytes assessed UCP1 after 3–8 h [46], [47], whereas studies using primary human brown or beige adipocytes reported both increased [48] or decreased [49] *UCP1* expression after 24 h. Thus, we cannot exclude transient effects at earlier time points. The lack of effect on *UCP1* expression does not imply that there is no effect on thermogenesis as there are other mechanisms of thermogenesis such as ATP-dependent futile cycling [50], [51], [52], [53], [54]., Moreover, we showed that the treatment with exercise-conditioned serum of endurance-trained participants increased the catecholamine-induced lipolysis in beige adipocytes but not intracellular cAMP levels. Thus, the serum might influence the lipolytic responsiveness but not thermogenic activation of beige adipocytes. Importantly, these in vitro experiments only assess the effects of circulating serum factors and cannot reproduce neural regulation, local noradrenaline release, tissue perfusion or the integrated hormonal environment of adipose tissue in vivo. Therefore, the absence of serum-induced *UCP1* expression should not be interpreted as evidence for the absence of adipose tissue thermogenesis in vivo.

This study has several limitations. First, we did not obtain WAT biopsies to assess *UCP1* expression in vivo. Repeated adipose tissue biopsies were considered too invasive for healthy participants and would likely have reduced recruitment and compliance. Instead, we assessed systemic and biochemical markers that provide indirect evidence of sympathetic activation and adipose tissue remodelling. Second, we have only measured noradrenaline but no other regulators of WAT browning. Third, the sample size was too small to permit exploratory sex-stratified analyses. Finally, no a priori power calculation was performed. The sample size was based on feasibility, considering participant recruitment, the resource-intensive intervention, and the extensive physiological and molecular assessments. Consequently, the study may have been insufficiently powered to detect small intervention effects, and the null findings should be interpreted accordingly.

In conclusion, six weeks of thrice-weekly fartlek-style cycling induced marked cardiorespiratory and metabolic adaptations but did not alter cold-induced oxygen uptake or the ability of conditioned serum to stimulate *UCP1* expression in human beige adipocytes. An exploratory analysis indicated an attenuated systemic noradrenaline response to cold following training, the physiological relevance of which requires further investigation.

The following are the supplementary data related to this article.

Supplementary material

## CRediT authorship contribution statement

**Alexander Braunsperger:** Writing – review & editing, Writing – original draft, Visualization, Validation, Software, Project administration, Methodology, Investigation, Formal analysis, Data curation, Conceptualization. **Lara Becker:** Project administration, Methodology, Investigation, Conceptualization. **David P. Camargo:** Investigation. **Sidney Radsak:** Investigation. **Magdalena Paintner:** Investigation, Data curation. **Dimitrios Kossenakis:** Investigation. **Xianyu Song:** Investigation. **Anna Kraus:** Investigation. **Alina Birnkammer:** Investigation. **Sebastian Mesmer:** Investigation. **Joshua Stengel:** Investigation. **Lukas Frank:** Investigation. **Svenja Kuger:** Investigation. **Tobias Mather:** Investigation. **Enni Peltola:** Investigation. **Marcel Könen:** Investigation. **Kirsten Liebetanz:** Investigation. **Jule Zorn:** Investigation. **Meri De Angelis:** Resources, Methodology, Investigation. **Martin Hrabe de Angelis:** Resources, Methodology, Investigation. **Dominik Tischer:** Writing – review & editing, Methodology, Investigation. **Ana Soriano-Arroquia:** Writing – review & editing, Resources, Investigation. **Anastasia Georgiadi:** Resources, Methodology, Investigation. **Martin Schönfelder:** Writing – review & editing, Writing – original draft, Visualization, Validation, Supervision, Project administration, Methodology, Investigation, Conceptualization. **Henning Wackerhage:** Writing – review & editing, Writing – original draft, Visualization, Validation, Supervision, Project administration, Methodology, Investigation, Funding acquisition, Conceptualization.

## Funding

H.W., M.S., A.G., A.S.A., D.T., and A.B. are funded by DFG (Deutsche Forschungsgemeinschaft/ German Research Association) within BATenergy (CRC/TRR333, 450149205).

## Declaration of competing interest

The authors declare that they have no known competing financial interests or personal relationships that could have appeared to influence the work reported on this paper.

## Data Availability

The datasets used and/or analyzed during the study are available from the corresponding author upon reasonable request.
